# Genetic and spectrally distinct *in vivo *imaging: embryonic stem cells and mice with widespread expression of a monomeric red fluorescent protein

**DOI:** 10.1186/1472-6750-5-20

**Published:** 2005-07-04

**Authors:** Jonathan Z Long, Chantal S Lackan, Anna-Katerina Hadjantonakis

**Affiliations:** 1 Developmental Biology Program, Sloan-Kettering Institute, New York, NY 10021, USA

## Abstract

**Background:**

DsRed the red fluorescent protein (RFP) isolated from *Discosoma sp. *coral holds much promise as a genetically and spectrally distinct alternative to green fluorescent protein (GFP) for application in mice. Widespread use of DsRed has been hampered by several issues resulting in the inability to establish and maintain lines of red fluorescent protein expressing embryonic stem cells and mice. This has been attributed to the non-viability, or toxicity, of the protein, probably as a result of its obligate tetramerization. A mutagenesis approach directing the stepwise evolution of DsRed has produced mRFP1, the first true monomer. mRFP1 currently represents an attractive autofluorescent reporter for use in heterologous systems.

**Results:**

We have used embryonic stem cell-mediated transgenesis to evaluate mRFP1 in embryonic stem cells and mice. We find that mRFP1 exhibits the most spatially homogenous expression when compared to the native (tetrameric) and variant dimeric forms of DsRed. High levels of mRFP1 expression do not affect cell morphology, developmental potential or viability and fertility of animals. High levels of widespread mRFP1 expression are maintained in a constitutive manner in embryonic stem cells in culture and in transgenic animals. We have used various optical imaging modalities to visualize mRFP1 expressing cells in culture, in embryos and adult mice. Moreover co-visualization of red, green and cyan fluorescent cells within a sample is easily achieved without the need for specialized methodologies, such as spectral deconvolution or linear unmixing.

**Conclusion:**

Fluorescent proteins with excitation and/or emission profiles in the red part of the visible spectrum represent distinct partners, or longer wavelength substitutes for GFP. Not only do DsRed-based RFPs provide a genetically and spectrally distinct addition to the available repertoire of autoflorescent proteins, but by virtue of their spectral properties they permit deeper tissue imaging. Our work in generating *CAG::mRFP1 *transgenic ES cells and mice demonstrates the developmental neutrality of mRFP1 in an organismal context. It paves the way for the use of DsRed-based monomeric RFPs in transgenic and gene targeted approaches which often necessitate spatially and/or temporally restricted reporter expression. Moreover animals of the *CAG::mRFP1 *transgenic strain serve as a source of RFP tagged tissue for the derivation of cell lines and explant, transplant and embryo chimera experiments.

## Background

GFP and many of its spectral variants have found widespread use as genetically encoded indicators in cell biological applications, including investigating protein expression, localization and interactions [1]. They have also been used for marking cells in complex tissues in chimeras, and for tagging gene expression in targeted or transgenic regimes in whole embryos, adult animals, explants and transplants [2,3]. In addition to the many cell biological benefits provided by increasing the number of genes encoding fluorescent protein species and extending the spectrum of available colors to longer (red) wavelengths of the spectrum, access to red fluorescent protein (RFP) variants would offer benefits for genetically and spectrally-distinct imaging of multiple cell populations in complex tissues [2,4].

All genetically-encoded coelenterate-derived fluorescent proteins cloned to date display some form of quaternary structure [5]. The weak tendency of *Aequorea victoria *green fluorescent protein (GFP) to dimerize has not impeded its use in exogenous systems. In contrast the obligate tetramerization of the most popular RFP DsRed [6], isolated from *Discosoma sp.*, has impeded its widespread use in mice [3]. In addition to its obligate tetramerization, the evolution of DsRed to a generally applicable and robust tool has been hampered by several critical problems, including its slow and incomplete maturation [7].

An alternative approach to overcoming the shortcomings of DsRed has been to continue the search for DsRed homologues in sea corals and anemones [8-10]. This approach has yielded several novel red-shifted proteins, however the more fundamental problem of tetramerization still exists and needs to be overcome. Therefore it appears likely that a mutagenesis approach focused on a single tetrameric RFP and designed to sequentially render it dimeric and then monomeric while retaining fluorescence quantum yield and extinction coefficient, is key to achieving progress in the field [11].

Attempts, to mutagenize the DsRed coding sequence to improve the rate and or extent of maturation have resulted in several variants including DsRed2. Unfortunately DsRed2 has provided only modest improvements and has not effectively eradicated the apparent 'toxicity' of DsRed [12]. Additional engineered variants, including DsRed.T1, DsRed.T3 and dimer2 have superceded DsRed2, as they have faster more complete maturation [11,13]. However, since these proteins still form obligate dimers, the heterogeneity of protein localization (observed as 'clumps' within cells) has not been overcome [13,14].

Although many dimeric DsRed variants have found widespread use in other organisms including yeast, worms, fruitflies and zebrafish, several investigators, including ourselves, have not previously been successful in using them in mice [3]. Recently mice with widespread conditionally activated DsRed.T3 expression have been reported [15]. Interestingly though high-resolution imaging of embryonic stem cells and cells in chimeras that exhibit constitutive expression of DsRed.T3, or of related dimeric DsRed variants, reveals heterogeneous expression within cells, and in particular, punctate staining in a perinuclear region, possibly the Golgi (our unpublished observations). Although reduced in intensity, this subcellular localization is reminiscent of that observed with tetrameric DsRed variants. It is therefore probable that dimeric DsRed variants are not the optimal starting point for RFPs used in mouse transgenic and gene targeting regimes.

Moreover the available dimeric DsRed variants have not resolved the issue of incorporating RFPs into protein fusions that can, for example, be used for *in vivo *imaging at subcellular resolution [12], therefore necessitating the development of monomeric RFPs which may provide improved performance and yield developmental neutrality when incorporated into fusions.

The first true monomer designated mRFP1 (monomeric RFP 1), was reported a few years ago [11]. The stepwise evolution of the DsRed sequence to generate mRFP1 involved mutations that first increased the speed of maturation, then mutations which resulted in the breaking of each subunit interface, and then a further round of mutations resulting in restoration of fluorescence. This directed evolution of an RFP to a monomer resulted in the introduction of a total of 33 amino acid substitutions [11]. mRFP1 has been reported to participate in, but not impair, the function of various fusion proteins in cases where both the tetrameric and dimeric DsRed variants were unable to do so. This makes mRFP1 attractive for use in mice both in its native form and as part of a fusion protein.

Furthermore, mRFP1 has recently served as the template for further mutagenesis directed at improving its performance in N-terminal protein fusions, fluorescence quantum yield and extinction coefficient, photostability and/or in providing a variety spectral variants [16]. This has resulted in the next generation of monomers, which include a battery of spectrally-distinct DsRed variants spanning the spectrum from green (mHoneydew), through yellow (mBanana) to a range of reds (mOrange, mStrawberry, mCherry). Therefore monomeric RFPs based on DsRed currently represent the most attractive genetically-encoded red fluorescent proteins for use in mice. However, the developmental neutrality and fluorescent intensity of monomeric RFPs has not been investigated *in vivo *in transgenic or gene targeting applications mice.

## Results

We have investigated the expression and germline transmission of native mRFP1 in embryonic stem cells and mice. This is an essential prerequisite to using the available suite of DsRed-derived monomeric RFPs in fusions as reporters for high-resolution *in vivo *imaging, and in particular, as fusion proteins exhibiting subcellular localization and acting as segmental markers of 3-dimensional space.

As a first step we cloned the *mRFP1* gene into a vector permitting widespread expression in a variety of cells types in culture and *in vivo *in mice. The *mRFP1 *coding sequence was engineered to contain a Kozak consensus sequence at its 5' end and subsequently introduced into *pCAGGS* a vector utilizing the chicken beta actin promoter and *SV40 *immediate early enhancer combination, designed to drive high-level constitutive gene expression in ES cells, embryos and adult mice [17,18]. Standard protocols were used to establish stable *CAG::mRFP1 *transgenic lines of ES cells constitutively expressing mRFP1.

Fluorescent colonies (*CAG::mRFP1 *transgenic) were identified and picked under an epifluorescence stereo dissecting microscope. Clones were passaged in 96-well plates, and scored for the maintenance and extent of red fluorescence with maintenance in culture. Clones that failed to meet these criteria were discarded from further analysis. Thereafter only clones exhibiting robust transgene expression *in vitro *under both stem cell and differentiation conditions were further analyzed for level and heterogeneity of red fluorescence by flow cytometry as described previously for GFP-variants [19]. Only those clones exhibiting high levels of homogenous expression were selected for further analysis. Sustained strong homogenous red fluorescence and retention of normal ES cell morphology when grown on either gelatin-coated plates or mouse embryo fibroblast feeders was a prerequisite feature of these cells

**Figure 1:**
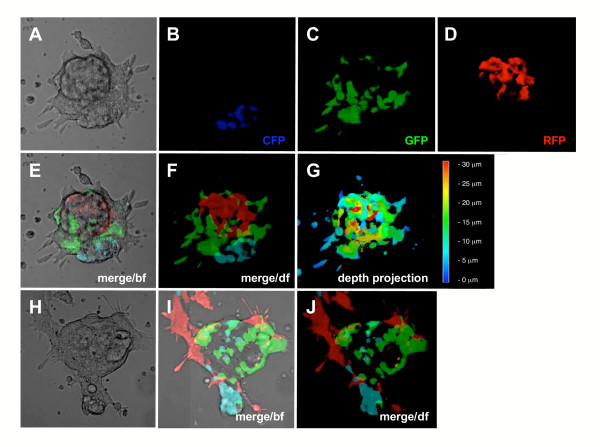
**Co-visualization of multiple fluorescent proteins in mouse embryonic stem (ES) cells**. Mixed colony of embryonic stem (ES) cells comprised of wild type (untagged) cells, *CAG::ECFP *transgenic cells exhibiting widespread expression of ECFP, *CAG::EGFP *transgenic cells exhibiting widespread expression of EGFP and *CAG::mRFP1 *transgenic cells exhibiting widespread expression of mRFP1. Bright field image (A), CFP channel (B), GFP channel (C), RFP channel (D), merge of all three fluorescent channels overlayed on the bright field image (E), merge of all three fluorescent channels (F), color-coded depth projection of the three fluorescent channel merge with the color-coded scale shown on the right of the image (G). A second mixed transgenic ES cell colony with bright field image (H), merge of three fluorescent channel merge overlayed on the bright field image (I) and dark field three fluorescent channel merge (J). In all panels except G ECFP fluorescence is shown in blue, EGFP fluorescence is in green and mRFP1 fluorescence is in red. bf, bright field; df, dark field.

We then co-cultured three transgenic ES cell lines expressing different fluorescent proteins, namely; *CAG::ECFP*, *CAG::EGFP *and *CAG::mRFP1 *along with wild type (non-fluorescent) ES cells, and then visualized fluorescence in resulting chimeric colonies (Fig. 1). The different fluorophores could easily be distinguished both with standard filter sets using epifluorescence optics and confocal microscopy, without the need for special image processing such as spectral deconvolution or linear unmixing [20]. We also noted that comparable levels of fluorescence were produced as all three color variants produced fluorescent signal within the same dynamic range.

Having established the neutrality of widespread mRFP1 expression in ES cells, we went on to use ES cell mediated transgenesis through the generation of germline transmitting chimeras to introduce the *CAG::mRFP1 *transgene into mice. As a first step to test the extent of expression of the transgene in embryos, we generated 4n (tetraploid) wild type <-> mRFP1 ES cell derived chimeras [21], exactly as described previously [3]. Resulting, completely ES cell-derived embryos exhibited widespread mRFP1 expression, indicating that the level of expression was sufficiently strong to be visualized, that the transgene was not silenced and that development was able to proceed normally to midgestation (data not shown). To produce germline transmitting chimeric adult animals, we next generated diploid wild type <-> mRFP1 ES cell chimeras that were allowed to go to term. The *CAG::mRFP1 *transgene was transmitted to F1 offspring in a Mendelian fashion, suggesting that widespread mRFP1 expressing is compatible with normal development and fertility. Two ES cell lines were taken germline and shown to produce an equivalent intensity and range of fluorescence, therefore data from only one line are shown.

**Figure 2:**
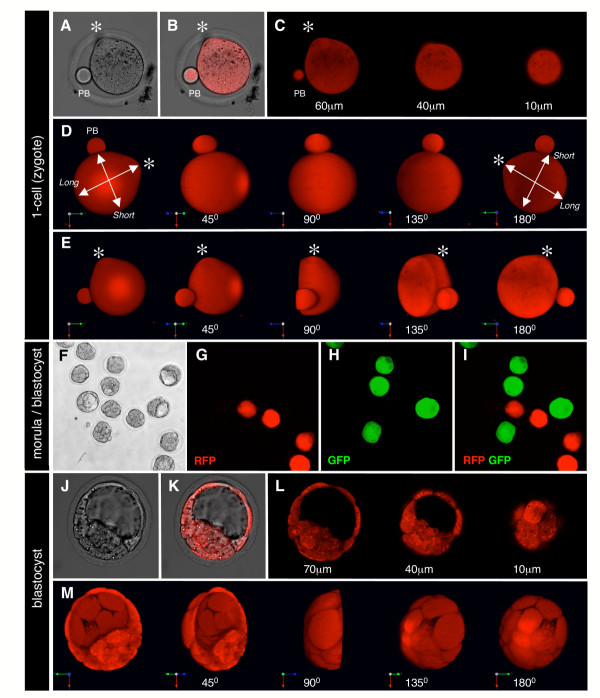
**RFP expression in *CAG::mRFP1 *preimplantation stage embryos**. Single *CAG::mRFP1 *Tg/+ zygote including the second polar body (A–E). A single *x-y *section taken from a *z*-stack, bright field image (A), overlay single confocal section of red fluorescence and bright field (B), red fluorescence channel only (left panel in C). Representative *x-y *sections taken from the same *z*-stack that was used to render volumes shown D and E. Rendered *z*-stack (3D reconstruction) of the whole zygote and second polar body (PB) shown in the previous panels and rotated through 180 degrees counter-clockwise (D). Rendered *z*-stack (3D reconstruction) of a computationally bisected zygote shown in the previous panels and rotated through 180 degrees counter-clockwise (E). Note that the zygote is not spherical, it has a clear short and long axis (lines with arrows), and the fertilization cone resulting from the site of sperm entry is also clearly evident as a protrusion (asterix). Non-transgenic, *CAG::EGFP *Tg/+, *CAG::mRFP1 *Tg/+ embryos recovered at E3.0 and representing compacted morulae through to blastocyst stages (F–I). Bright filed (F), red fluorescence channel (G), green fluorescence channel (H) and green and red fluorescence channel overlay (I). Single *CAG::mRFP1 *Tg/+ blastocyst (J–M). A single *x-y *section taken from a *z*-stack, bright field image (J), overlay single confocal section of red fluorescence and bright field (K), red fluorescence channel only (left panel in L). Representative *x-y *sections taken from the same *z*-stack that was used to render the volume shown in M. Rendered *z*-stack (3D reconstruction) of a computationally bisected blastocyst and rotated through 180 degrees counter-clockwise (M). Note that individual cells of the trophectoderm can be distinguished. The RGB colored vector on the bottom left of the 3D reconstruction rotations depicts the *x*-axis in green, *y*-axis in red and *z*-axis in blue.

Wide field epifluorescent and laser scanning confocal microscopy was used to image this constitutively expressed transgene reporter in preimplantation stage mouse embryos hemizygous (Tg/+) for the *CAG::mRFP1 *transgene (Fig. 2 and additional files). Such non-invasive visualization in living preparations allowed us to acquire high-magnification, sequential optical sections (*z*-stacks) that were used to generate high-resolution anatomical, volumetric images of embryos. To do this, stacks of sequential optical sections were computationally reconstructed into 3-dimensional (3D) projections. This methodology was used to generate 3D image sets, and is illustrated here by imaging whole mouse embryos at the 1-cell stage and blastocyst stage. These data sets can be computationally manipulated in various ways including for the visualization of individual *xy *slices from a *z-*stack (Fig. 2B,C,K and 2L and additional files), or of rendered images from the full (Fig. 2D), or partial *z*-stack (Fig. 2E and 2M). It should be noted that even using epifluorescence imaging *CAG::mRFP1 *embryos were clearly distinguished from stage-matched *CAG::EGFP *embryos (Fig. 2G–I).

**Figure 3:**
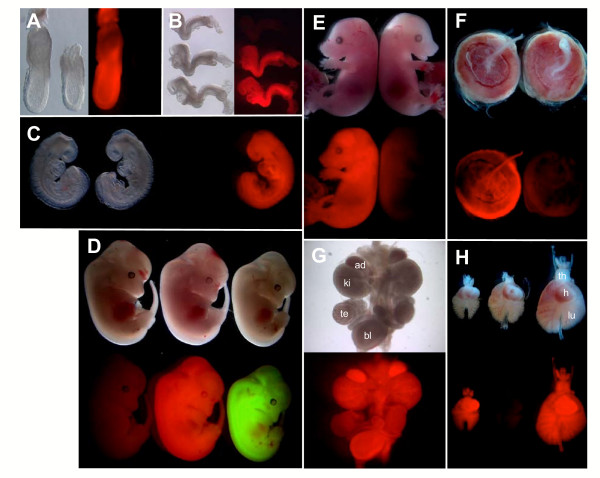
**RFP expression in *CAG::mRFP1 *postimplantation embryos**. Bright field and dark field epifluorescent images of CAG::mRFP1 Tg/+ embryos and non-transgenic littermates at E6.5 (A), E7.75 (B), E8.75 (C). CAG::mRFP1 Tg/+ embryos, CAG::EGFP Tg/+ embryos and non-transgenic littermates at E11.5 (D). E15.5 CAG::mRFP1 Tg/+ fetuses and non-transgenic littermates at (E) demonstrating widespread homogenous red fluorescence throughout later development in whole embryos, dissected embryonic tissues (G and H) and extraembryonic tissues including the placenta (F). (H), cardiothoracic organs from three embryos of different ages, E13.5 (left), E14.5 (center) and E15.5 (right), only two of which are hemizygous for the transgene). Ad, adrenal gland; bl, bladder; h, heart; ki, kidney; lu, lung; te, testis; th, thymus.

Wide field microscopic imaging of later stage hemizygous embryos illustrated the robust, homogenous and widespread expression of mRFP1 from early postimplantation, embryonic day (E) 6.5 (Fig. 3A) to later fetal stages in both the embryo proper and extraembryonic lineages, including the placenta (Fig. 3E–F). Dissection of organs from fetuses confirmed widespread red fluorescence in *CAG::mRFP1/+ *organs contrasted with a lack of signal observed in non-transgenic littermates (Fig. 3G and 3H).

**Figure 4:**
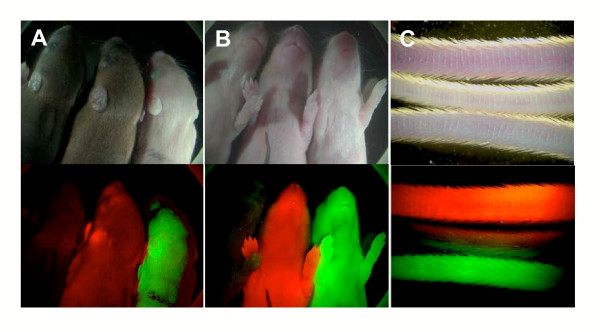
**Transgenic *CAG::mRFP1 *mice can be distinguished from *CAG::EGFP *animals**. Macroscopic images of non-transgenic, *CAG::mRFP1 *Tg/+ and *CAG::EGFP *Tg/+ newborn (P5) mouse pups demonstrating that red fluorescence can clearly be distinguished from green fluorescence using conventional epifluorescent illumination and macroscopic observation. Dorsal view (A), and ventral view (B), high magnification of tails of 3 month old animals (C). Inspection of fluorescence in the tails is the method used for routine genotyping of these strains.

Examination of newborn animals revealed strong widespread expression of mRFP1 in the skin of hemizygous *CAG::mRFP/+ *transgenics and demonstrated that this red fluorophore can be distinguished from green fluorescence observed in *CAG::EGFP/+ *transgenics, and from non-transgenic littermates by standard macroscopic visualization using standard filter sets and epiflourescent excitation (Fig. 4).

**Figure 5:**
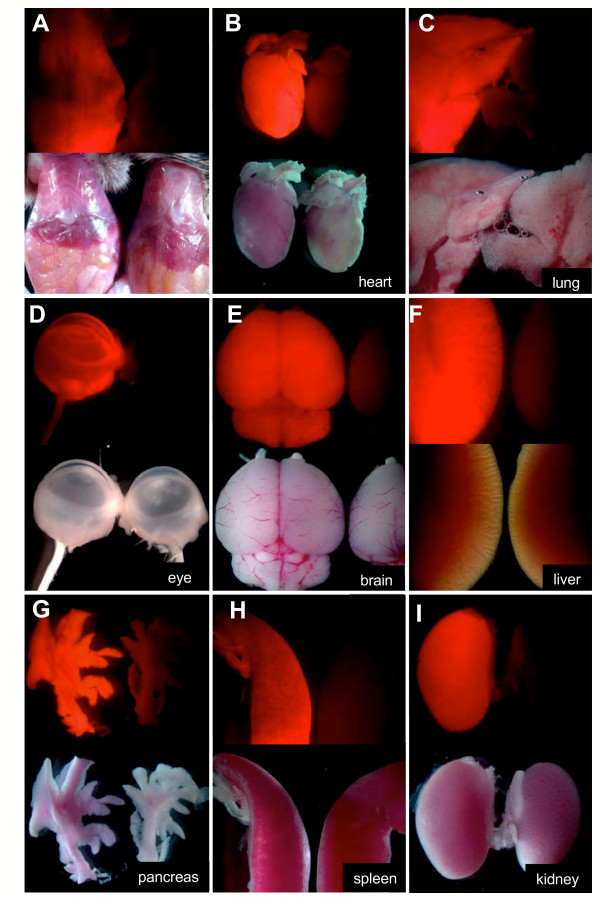
**Widespread RFP expression in adult organs**. Panels of bright field and corresponding dark field epifluorescent images of organs taken from a 4 week old *CAG::mRFP1 *Tg/+ mouse and a non-transgenic littermate. Peritoneum (A), heart (B), lung (C), eye (D), brain (E), liver (F), pancreas (G), spleen (H) and kidney (I). In addition to the fluorescence observed under epifluorescent illumination, RFP expressing tissues exhibit a pink color under bright field illumination. This is particularly evident in panels A, B, C, E and G. This allows for genotyping of newborn pups (by virtue of their pink color) in the absence of fluorescence illumination.

Further analysis of various adult organs, including the peritoneum, heart, lung, eye, brain, liver, pancreas, spleen and kidney, that were freshly obtained from *CAG::mRFP1/+ *adult animals revealed robust and widespread fluorescence (Fig. 5), as has been reported for animals expressing GFP-based fluorescent proteins under the regulation of the CAG promoter. We also noted that newborn pups, or bald skin and tissues expressing mRFP1, exhibit a pink coloration under normal light when compared to non-transgenic or *CAG::EGFP *transgenics [18]. This is particularly evident in albino animals (tails in Fig. 4C), and in unpigmented organs such as the brain and pancreas (Fig. 5E and 5G). We believe this results from mRFP1 being a red fluorphore with a spectrum closer to the visible range, so that it can be visualized as a pink pigmentation under daylight or bright field conditions. Consequently, newborn *CAG::mRFP1 *pups can be genotyped based on their pink pigmentation, alleviating the need to image them under epifluorescent conditions.

Moreover we succeeded in breeding the *CAG::mRFP1 *transgene to homozygosity. Homozygous animals retained developmental potential and therefore colonies of the strain are routinely maintained in the homozygous state. To date, we have observed no noticeable reduction in fluorescence or fertility for over four generations on both ICR outbred and 129 inbred backgrounds. From routine daily observations no behavioral differences are distinguished between *CAG::mRFP1 *animals and wild type age matched animals, though no behavioral tests have been implemented.

## Discussion

We have established the applicability of monomeric RFPs as an alternative spectrally-distinct genetically-encoded fluorescent reporter to GFP and its variants. Now monomeric DsRed variants can be used *in vivo *in mice along side GFP variants to differentially label single cells or different populations of cells.

Advances in optical imaging modalities, and the development of *ex vivo *embryo or explant culture systems, have evolved alongside the increasing availability of genetically-encoded fluorescent proteins permitting dynamic *in vivo *imaging of living specimens. Two major limitations in the application of genetically-encoded fluorescent protein technology in mice, have been the lack of fluorescent proteins in the longer wavelength (red) part of the spectrum, and the absence of a fluorescent protein that is genetically distinguishable from GFP. DsRed, the coelenterate derived RFP, has offered the potential to solve both these problems, but original variants, which function as obligate tetramers and dimers, have exhibited 'toxicity', limiting their use in heterologous systems.

Our work demonstrates the developmental neutrality of mRFP1, the first true monomeric RFP, in ES cells and mice. The strain of mice we have generated exhibits widespread mRFP1 expression, providing a novel reagent for live imaging of red fluorescent cells in a genetically tractable mammalian model organism. *CAG::mRFP1 *animals represent a resource for analyzing development and disease in mouse embryos and adults. They can also be used as tagged populations of cells in chimeras, in addition to transplantation and cell isolation experiments. Moreover RFP cells can easily be distinguished from GFP, or GFP spectral variant expressing cells (including ECFP and EYFP) by both imaging using standard optics, and molecular analyses, due to the different genetic origin.

To date all fluorescent proteins isolated from diverse anthozoan species suffer from obligate tetramerization and will require efforts similar to the evolution of mRFP1 to produce widely useful tools. Our demonstration of the developmental neutrality of widespread mRFP1 expression paves the way for the incorporation of monomeric RFPs into mouse transgenic and gene targeting approaches [2]. Our data on mRFP1 suggest that its mutagenized derivatives will also be amenable to use in mice. If so, there should now be at least six new spectral variant monomeric FPs based on DsRed that can be used in mice. Key applications of the proliferation of spectral variant FPs will include discriminating different cells, transcriptional activities and/or fusion proteins.

## Conclusion

Our work demonstrates the use of monomeric RFPs in mice and provides the basis for future efforts designed to incorporate RFPs into protein fusions that can be used as spectrally-distinct subcellularly-localized tags permitting high-resolution live imaging *in vivo *[12,22]. The ongoing development of mouse strains expressing subcellularly-localized protein fusions incorporating RFPs, and contrasting with GFP-variants will be essential for visualizing 4-dimensional (3-dimensions over time) anatomy and tracking cell position, morphology and behavior *in vivo*.

## Methods

### Vector construction

The mRFP1 coding sequence was amplified by PCR using primers 5'RFP (5'-cgtagaattcgccaccaatggctagcatgactgg) and 3'RFP (5'-gcacgaattcgggcgccggtggagtggcggcc) using *Pfx *Polymerase (Invitrogen). The resulting product was cloned into the *Eco*RI site *of pCAGGS *to generate *pCX-mRFP1*. Transient transfection of Cos-7 cells using Fugene 6 Transfection Reagent as per manufacturer's recommendations (Roche) was used to evaluate *pCX-mRFP1*, and verify that it produced robust red fluorescence.

### Generating transgenic ES cells

Transgenic ES cell lines constitutively expressing mRFP1 were generated by co-electroporation of *Sal*I linearized *pCX-mRFP1 *construct and a circular *PGK-Puro-pA *plasmid conferring transient puromycin resistance. Puromycin selection was carried out exactly as described previously [18]. Thereafter cells were passaged according to standard protocols.

### Mouse breeding

Two lines of *CAG::mRFP1 *ES cells were used for chimera generation by injection into C57BL/6 blastocysts using standard procedures [23]. Chimeras were mated to outbred ICR and inbred 129/Tac mice (Taconic, Germantown, NY) for germline transmission and subsequent maintenance of the lines. After germline transmission, both transgenes were bred to, and maintained at, homozygosity, suggesting that the sites of transgene integration were not perturbing essential gene function and that high levels of protein expression were non-toxic. Animals from both lines retained widespread homogenous fluorescence in all subsequent generations tested and therefore data was pooled.

### Embryo collection

Preimplantation embryos were recovered in M2 media and subsequently cultured under oil in a tissue culture incubator gased at 5% CO2 in KSOM media. Postimplantation embryos and organs were dissected either in HEPES buffered DMEM containing 10% fetal calf serum or PBS containing 0.1% BSA, and cultured in media comprising 50% rat serum, 50% DMEM/F12 supplemented with L-glutamine.

### Image acquisition

Also although mRFP1 has been shown to have a lower extinction coefficient, quantum yield, and photostability than native DsRed or DsRed.T3, mRFP1 has been shown to mature over ten fold faster, so that it shows similar brightness in living cells. In addition, the excitation and emission peaks of mRFP1 (584 and 607 nm respectively) are 25 nm red-shifted from DsRed, which should confer greater tissue penetration and spectral separation from autofluorescence and other fluorescent proteins. All images presented in the figures are of living embryos or freshly dissected (unfixed) tissues maintained under physiological conditions. Wide-field images were acquired with an AxioCam MRc camera attached to a Leica MZ16FA stereo-dissecting microscope or a Zeiss Axiovert 200M inverted microscope equipped with epifluorescent illumination using appropriate filter sets (Chroma). Laser scanning confocal data was acquired in single-track mode using a Zeiss LSM510 META on a Zeiss Axiovert 200M. Fluorophores were excited with a 405 nm diode laser (ECFP), 488 nm Argon laser line (EGFP) and a 543 nm HeNe laser (mRFP1). Objectives used were a C-apochromat 40×/NA1.2, plan-apochromat 20×/0.75 and a fluar 5×/0.25. Confocal images were acquired as *z*-stacks comprising sequential optical *x*-*y *sections taken at 1–2 μm *z*-intervals.

### Image processing

Raw data was processed using Zeiss AIM software (Carl Zeiss Microsystems at ), and Volocity (Improvision at ). Re-animation of data to generate movies of time-lapses or rotations was performed using QuickTime Pro (Apple Computer, Inc at ).

## Authors' contributions

JZL and CSL carried out the preimplantation embryo experiments including all imaging and the generation of the aggregation chimeras. AKH planned the study, carried out the experiments in ES cells and postimplantation embryos, and wrote the manuscript.

## Footnote

The *CAG::mRFP1 *mice described in this paper will be made available from the Jackson Laboratories Induced Mutant Resource () as stock number #5645.

## Supplementary Material

Additional File 1 Sequential *x-y *images of a *z*-stack taken through a *CAG::mRFP1 *Tg/+ zygote (1-cell stage). The first part of the sequence includes bright field (DIC) images merged with the red fluorescence channel, while the second part depicts only the red fluorescence channel. Click here for file

Additional File 2 3D reconstruction and rotation of the complete raw data set shown in Movie 1 depicting a view of a full zygote. The sequence depicts the rendered image being rotated around each axes (*z*, *y *and *z*). The RGB colored vector on the bottom left depicts the *x*-axis in green, *y*-axis in red and *z*-axis in blue. Click here for file

Additional File 3 3D reconstruction and rotation of half the raw data shown in Movie 1 depicting a view of a computationally bisected zygote. The sequence depicts the rendered image being rotated around each axes (*z*, *y *and *z*). The RGB colored vector on the bottom left depicts the *x*-axis in green, *y*-axis in red and *z*-axis in blue. Click here for file

Additional File 4 Sequential *x-y *images of a *z*-stack taken through a CAG::mRFP1 Tg/+ blastocyst stage embryo. The first part of the sequence includes bright field (DIC) images merged with the red fluorescence, while the second part depicts only the red fluorescence channel. Click here for file

Additional File 5 3D reconstruction and rotation of the complete raw data set shown in Movie 4 depicting a view of half a blastocyst. The sequence depicts the rendered image being rotated around each axes (z, y and z). The RGB colored vector on the bottom left depicts the x-axis in green, y-axis in red and z-axis in blue. Click here for file
